# Hearing aid trial periods: Audiologists’ thoughts and practices in South Africa

**DOI:** 10.1371/journal.pgph.0002552

**Published:** 2023-11-03

**Authors:** Angie Heliopoulos, Nomfundo Moroe

**Affiliations:** Department of Speech Pathology and Audiology, University of the Witwatersrand, Johannesburg, South Africa; Stellenbosch University, SOUTH AFRICA

## Abstract

The process of adjusting and becoming accustomed to hearing aids may be best facilitated by providing a hearing aid trial period. Globally, there are no standardised frameworks or regulations on the recommended hearing aid trial period. The main purpose of this study was to explore audiologists’ hearing aid trialing practices. A cross sectional study employed a quantitative, descriptive design to formulate the study. Ninety-five audiologists’ were recruited by means of a purposive sampling strategy. Data were collected through the use of an online survey via Google forms. A pilot study was conducted prior to the commencement of the main study in order to ensure reliability of the main study. The results indicated that two weeks was the most recommended duration of a trial period from audiologists’. Majority of audiologists’ (72.63%) offer hearing aid trials to their patients. Most audiologists’ who offer hearing aid trials choose to trial their patients with two different hearing aids. Findings revealed a need for trialing periods to become standard practice by audiologists’ when fitting hearing aids. Not enough audiologists’ are providing this service.

## Introduction

According to the World Health Organization (WHO), it is estimated that approximately 5% (432 million adults and 34 million children) of the world’s population experience a disabling hearing loss. This figure is expected to rise significantly by 2050, with around 1 in every 10 people, roughly 700 million individuals, projected to have a disabling hearing loss [1]. For adults aged 15 years and older, a disabling hearing loss is defined as a loss of hearing greater than 35 decibels (dBs) in the better hearing ear [1]. Interestingly, a significant majority of those affected, 80% reside in low and middle income countries such as Sub-Saharan Africa, South Asia, and Asia Pacific region [1]. Specifically, in South Africa, the prevalence of hearing loss is estimated to be much higher than reported 5% figure, making hearing loss the third highest reported disability alongside vision and physical disability [2]. The data used for estimating the population living with disabilities, including hearing loss, is based on outdated information such as the 2011 national census, which is over a decade old. Therefore, these reports may be unreliable and inaccurate in assessing the true extent of hearing loss in the country [2,3].

### Hearing aids uptake

Despite the high prevalence of hearing loss in the elderly population worldwide, the use and uptake of hearing aids is still poor [4,5]. A plethora of studies have investigated the reasons for poor uptake of hearing aids. These including hearing aids being uncomfortable, difficulty handling hearing aids, patients’ attitudes towards hearing loss and hearing aids, and poor understanding of the role of audiologists’ in supporting and counselling patients to promote hearing aid use [6–8].

Hearing aids have the potential to significantly improve an individual’s access to sound in various environments [9] providing several clinically- proven benefits to the wearer. These benefits include reducing listening effort, enhancing communication, slowing down cognitive decline and improving overall quality of life (QoL) [10]. When a person is initially fitted with a hearing aid, the duration and severity of their hearing loss determine the process needed in their management. in most cases, the hearing aid stimulates the ear-brain neural network system, with the sound being encoded at different stages of processing, including: the ear, brainstem, midbrain, and the cortex [11]. Consequently, the brain requires time to readjust and become familiar once again with the high and low frequency sounds of speech and environmental noise. To facilitate this adjustment process, it is recommended to provide individuals with a hearing aid trial period. During this trial period, the user is provided the opportunity to try out hearing aids before making a purchase, with the option to return or exchange them [12]. Typically, a trial period is conducted to allow the audiologist or hearing aid dispenser the opportunity to ensure a comfortable fit of the hearing aid, fine tune them for personalised sound quality, prevent feedback and enable the user to use the hearing aids in various listening environments [13]. At the conclusion of the trial period, the user has the option to retain the hearing aids, try out new hearing aid(s) as a trial or return the hearing aids [13]. Although literature suggests a minimum duration of 30 days for a hearing aid trial [13,14], there is currently no evidence-based practice to support this specific time period.

### Standardization of hearing aid trials

Globally, the practice of conducting hearing aid trial seems to be an ‘up-in-the-air’ process, lacking standardised frameworks or regulations regarding the recommended duration of the trial period. This absence of standardised guidelines on the hearing aid trial period as well as the number hearing aids available for trial leads to inconsistency across different parts of the world [15].

As mentioned earlier, hearing aid users need several weeks or more to acclimatize and ‘re-train the brain’ to identify certain frequencies and sounds. It is for this reason that in the United States, it is against the law to not offer trial periods to potential users as people with different types of hearing loss respond differently to hearing aids [12]. In countries such as South Africa, where there are no laws or regulations pertaining to hearing aid trial periods, some audiologists’ establish their own trial periods [12]. This indicates that audiologists’ are aware of the need to have policies or regulations governing the evidence-based practice when dispensing hearing aids to potential users. Currently, there are no international regulatory or governing bodies overseeing the sale and manufacturing of hearing aids. In the USA for instance, the sale of hearing aids is regulated by the Federal Trade Commission (FTC) and the Food and Drug Administration (FDA) [15]. According to these entities, any individual who feels they may struggle adapting to amplification should enquire about trialing or renting the hearing aids before making any decisions on purchasing them [15]. In South Africa, theoretically, the sale of hearing aids fall under the ambit of the South African Health Products Regulatory Authority (SAHPRA). SAHPRA is the regulatory authority responsible for ensuring the quality, safety, and efficacy of health products, including medical devices such as hearing aids [16]. Specifically, hearing aids are regulated by the Medicines Control Unit, under the Rule 9(1)(a)–an active medical device for therapy to administer energy to a patient, or exchange energy to or from a patient. Thus, a hearing aid is an example of a sound energy device. This document does not give guidance on the practise of providing hearing aid trials for audiologists’ or the warranty period of hearing aids.

A lack of standardised hearing aid trials may contribute to unintended consequences including:

- unequal access: some individuals may not have the opportunity to adequately assess the suitability and effectiveness of hearing aids before making a decision. This is particularly true for adults in lower socioeconomic settings, resulting in a lower prevalence of hearing aid use [17].
- limited evaluation time: without a standardized trial period, patients may have limited time to assess the benefits and limitations of hearing aids, potentially leading to dissatisfaction or suboptimal outcomes. One researcher argued that not all adults provided with hearing aids use them, wear them regularly, or are satisfied with them [18].
- Varied professional guidance: hearing aid trial periods often involve guidance and support from audiologists’ or hearing healthcare professionals. Without standardization, the level of professional guidance during the trial period may differ, potentially impacting the quality of fitting, counselling and adjustments made to optimize the hearing aid settings for individual users. According to Ismail et al, hearing healthcare professionals can exert an important impact on patient outcomes [19].
- Increased financial risk: hearing aids are expensive, and most insurance plans don’t cover routinely cover them, and in most cases, users typically pay for aids and fittings out of pocket [20]. Without standardized trial periods, hearing aids can be a significant financial if individuals make a decision to purchase without sufficient time to determine if the hearing aids meet their needs and preferences. This potentially leads to financial losses if the chosen hearing aid does not provide the desired outcomes.
- Quality Assurance: consistency across manufactures in describing the features of hearing aids enables users to compare features and not solely rely on audiologists’, and hearing aid dispensers for that information [21] Providing standardized Challenges with providing hearing aid trials in low and middle income countries.

Despite the high prevalence of disabling hearing loss in the African region, there is a significant shortage of audiologists’ to provide hearing healthcare services. Approximately 78% of countries in Africa have less than one audiologist per million population, whereas 52% of European countries have more than 10 audiologists’ per million [22]. This scarcity of human resources limits the capacity to diagnose and manage hearing loss using hearing technology, including the ability to offer hearing aid trial periods.

In South Africa the healthcare system is divided into public and private sectors. Around 84% of the population relies on public healthcare services, while 16% affords private healthcare [22,23]. As a result, the majority of the South African’s with a hearing loss depend on the public sector for their healthcare needs [24]. Moreover, one study reported that only 22% of audiologists’ are employed in the public sector [25,26]. This imbalance in the distribution of audiologists’ negatively impacts service delivery and patient accessibility to hearing healthcare services, including the provision of hearing aids, and the opportunity for hearing aid trial periods. Furthermore, the private sector in South Africa may face pressure to meet sales targets of hearing aids, as it operates in a more market-driven environment. On the other hand, the public sector is funded by the government and may not have the same pressure to prioritize hearing aid sales. This distinction potentially influences the availability and promotion of hearing aid trial period, as well as the overall approach to hearing healthcare. Collectively, these factors contribute to the challenges faced in providing adequate hearing healthcare services, including access to hearing aids and standardised trial periods, particularly within the public sector in South Africa. Addressing the shortage of human resource and promoting equitable access to hearing healthcare services are crucial for improving outcomes for individuals with hearing loss in low and middle income countries.

### Rationale

Evidence suggests that audiologists’ who offer hearing aid trials report a higher success rate in hearing aid sales and increased patient satisfaction [12,27]. A study by Knudsen et al [28] investigating hearing aid success and failures, it was found that 80% of participants who underwent hearing aid trials were satisfied with the process, resulting in a 60% hearing aid purchase rate in the study population [28]. This study appears to be the only one available on hearing trials, indicating a lack of large scale research on hearing aid trial periods globally. To fully understand the benefits of hearing aid trials, it is essential to start by investigating audiologists’ current practices in relation to hearing aid trials. Such research would provide valuable insights into the potential advantages of making trial periods a mandatory practice within rehabilitation process. Currently, there is a dearth of research in this area, globally. Thus this study aims to explore the practices of audiologists’ in providing hearing aid trials.

## Methods

### Research aims

1. To investigate audiologists’ practices related to hearing aid trials.

### The specific objectives of the study were

- To examine the practices used by audiologists’ for fitting hearing aids
- To analyse the approaches employed by audiologists’ during the hearing aid trialing process
- To determine the average number of hearing aids typically trialed by audiologists’
- To assess the average duration of a hearing aid trial conducted by audiologists’

### Research design

This was a two-phased explanatory sequential mixed methods cross-sectional study. Phase one was quantitative in nature and employed a questionnaire to capture audiologist’s practices in providing hearing aid trials. This was followed by a qualitative phase where interviews were conducted with hearing aid users to explore their experiences with undergoing a hearing aid trial. However, for the current study, we are reporting on Phase 1, exploring the practices of audiologists’ in providing hearing aid trials.

### Participants and sample size

Purposive sampling was used to specifically recruit audiologists’ practising in South Africa and registered with the Health Professions Council of South Africa (HPCSA). A prior sample size was not determined for this study due to the difficult in ascertaining the actual number of practicing audiologists’ in South Africa. The sample size could only be determined based on audiologists’ registered with the HPCSA or the professional organisations, which in most cases is inaccurate as some people may be registered but may not be practising or may be practising and not be registered with the professional association boards. Furthermore, this study was administered online, the researcher had to rely on the professional organisations to act as gatekeepers in distributing the survey link to potential participants. This provided a challenge as to the number of potential participants that could be reached. As such, it was difficult to estimate the number of participants to achieve an adequate statistical power and precision to ensure the study’s results are statistically meaningful and reliable. A sample of 95 audiologists’ was obtained (Table 1). Participants had to meet the necessary inclusion criteria to participate in the study. This included: audiologists’ currently registered with the HPCSA who were currently practicing at the time of the study in any institution within South Africa and its provinces.

**Table 1:** Descriptive characteristics of the study participants.

Variable	Category	n	%
Institution	Private practice	58	61.05
	Hospital	25	26.32
	Clinic	1	1.05
	School	2	2.11
	Rehabilitation center	1	1.05
	unspecified	8	8.42
Years of experience	0–5	36	38
	>5–10	17	18
	>10	34	36
	Community service	8	8

### Data collection method

For the first phase of the study, data were collected by means of an online survey conducted via Google Forms (S1 Appendix). Google Forms was chosen as it is freely accessible and user friendly. The survey was self-developed, consisting of 24 closed-ended questions and 13 open-ended questions. The open-ended questions consisted of an elaboration of some of the closed-ended responses. The survey was divided into six sections: background information, hearing aid characteristics, hearing aid trials, counselling, patient satisfaction, and brain maturation to hearing aids. The questionnaire was developed in English because it is widely used in professional communication and this study purposively recruited professionals. Based on the pilot study feedback, the survey completion time was five minutes on average. According to research, shorter surveys are more likely to be completed with a higher response rate than longer surveys [29]. Moreover, closed-ended responses have a quicker response duration. The majority of questions in this survey were closed-ended, thus, the time period to complete the survey was short.

### Pilot study

Three audiologists’ meeting the inclusion criteria were approached and requested to participate in the pilot phase of the study by direct invitation through the researcher’s network. The first audiologist was requested to assess the content, language and length of the survey. Feedback recommended: i) to include an option that ‘more than one option’ may be given on three of the survey questions; ii) Additionally, to add an option to elaborate on ‘no’ responses for three of the survey questions. Once the suggested corrections were made, the survey was then given to a second audiologist to provide feedback on the length and content of the survey. A change to the duration of the survey from ten minutes to five minutes was recommended as the completion time was quicker than anticipated by the researcher. Lastly, the content and questions were deemed straight forward. The third audiologist was recruited for the second phase of the study. Participants in the pilot study were required to meet the same inclusion criteria for the research study (as mentioned above). These two audiologists’ were excluded from the main study.

### Ethics statement

Ethical clearance was obtained from the University’s Human Research Ethics Committee (Medical) (Protocol number: M191054). Participants provided informed consent by clicking the ‘continue’ button on Google Forms after reading and understanding the information sheet. Participants were thus made aware that informed consent would be assumed once they proceeded with the study after reading the information sheet. All methods were carried out in accordance with relevant guidelines and regulations.

### Data collection procedure

After obtaining ethics clearance from the Human Research Ethics Committee (Medical) (Protocol number: M191054), professional organizations, namely the South African Association of Audiologists’ (SAAA) and South African Speech-Language-Hearing Association (SASHLA) were approached via email and requested to distribute the self-developed survey to audiologists’ on their emailing lists. Additionally, audiologists’ on social media platforms, such as Facebook groups and LinkedIn, were targeted. The first page of the survey consisted of the information letter, where the purpose, inclusion criteria and study requirements were detailed. Potential participants were advised that clicking on the start button at the end of the information letter meant they were giving consent to participate on the online survey. Upon clicking the start button, participants were directed to the survey question on Google Forms. Data collection took place over a period of six months between February and July 2020.

### Data analysis

Data were captured in Microsoft Excel and Microsoft Word. Both close-ended and open-ended questions were analysed quantitatively using descriptive analysis. Descriptive analysis is concerned with summarizing data with large amounts of numbers that could not otherwise be comprehended by looking at them. As such, frequency tables and graphs were used to summarize the data more comprehensively. This allowed the researcher to capture information and analyse it more efficiently.

## Results

### Hearing aid characteristics

From the 95 audiologists’ who participated in the survey, 94.74% (n = 90) provided hearing aid fittings as part of their services, while 5% (n = 5) do not fit hearing aids (Table 2). Table 2 below further indicates how majority of audiologists’ reported dispensing and fitting three or more hearing aid brands as part of their practice, providing their patients with a variety of options to choose from when selecting a hearing aid.

**Table 2:** Hearing aid fitting characteristics.

Provide hearing aid fittings							Do not provide hearing aid fittings	
**n**				**%**			**n**	**%**
**90**				94.74			5	5
**Number of hearing aid brands offered**								
**3+**		**2**			**1**			
**n**	**%**	**n**	**%**		**n**	**%**		
**72**	76	13	14		5	5		

Audiologists’ were asked how they choose the hearing aid for their patients or if patients choose their own hearing aids. The results revealed that 28.42% (n = 27) of audiologists’ choose the hearing aid for their patients based on their audiogram. On the other hand, 54.74% (n = 52) of audiologists’ choose the hearing aids, however, in consultation with their patients. This suggests that these audiologists’ do consider the patient’s needs as well as the situation (i.e. cost, appearance etc). The reasons that were given on which hearing aid to choose are indicated below (Table 3).

**Table 3:** Reasons for which hearing aid to choose.

Reasons	n	%
**Aesthetics and appearance chosen by the patient and technology range chosen by the audiologist.**	12	16.44
**Finances (medical aid vs private or government)**	16	21.92
**Hearing aid chosen according to the audiogram**	14	19.18
**Some patients are brand specific from previous experiences**	15	20.55
**Phone connectivity**	4	5.48
**Information provided**	12	16.44
**Total:**	**73**	**100**

**Fig 1:**
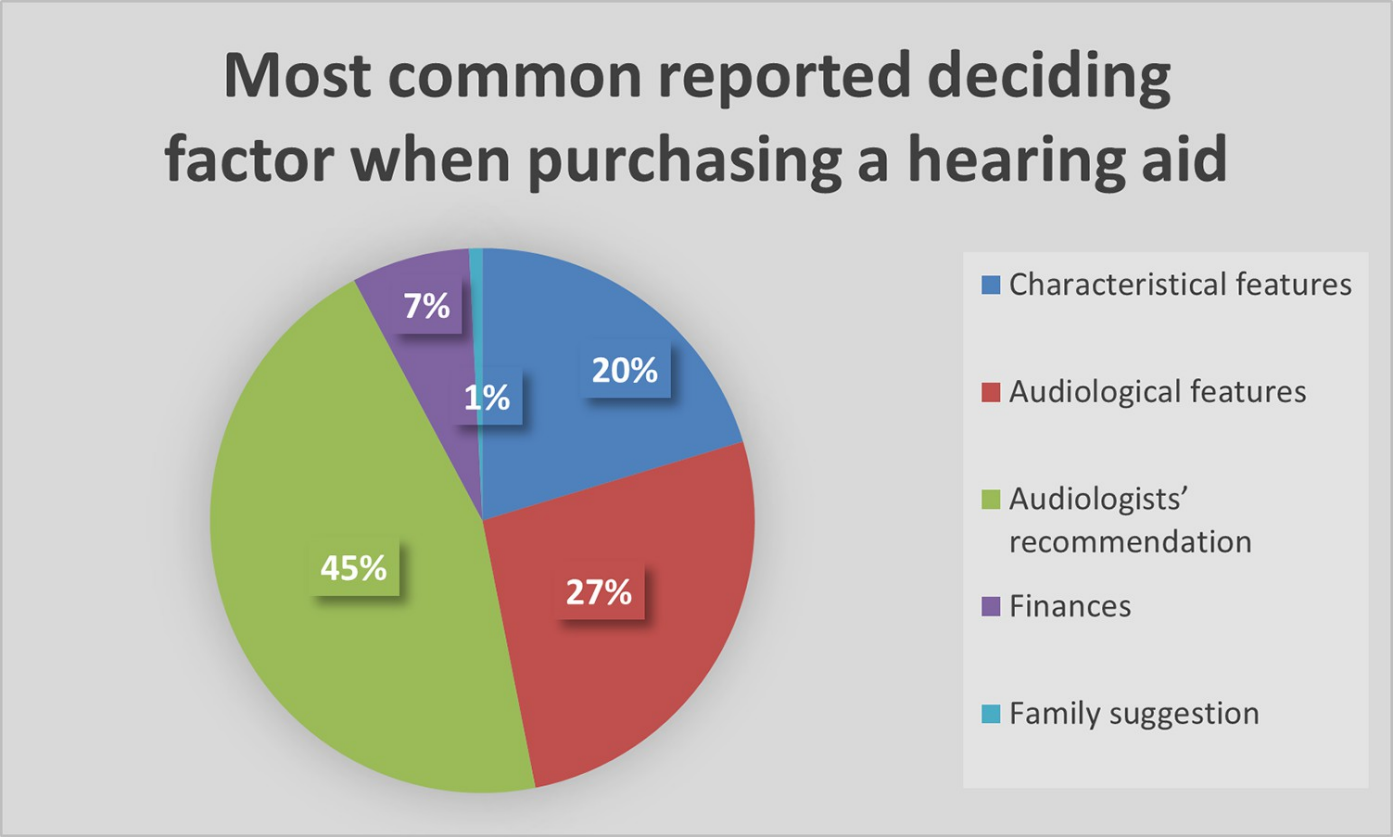
Most common reported deciding factor when purchasing a hearing aid.

Taking into account the patient’s hearing loss when choosing a hearing aid is one of the most important decisions within the hearing aid fitting process. Audiologists’ were further asked when choosing a hearing aid, what is the most common determining factor for the hearing aid user when deciding on which hearing aid to choose from. As seen in Fig 1 below, the audiologists’ recommendation as well as the audiological features of the hearing aid were highlighted as the most important factors.

### Return rates on hearing aids

The audiologists’ were asked to state possible explanations as to why some patients may return their hearing aids, with the option to choose more than one answer (Table 4). Top on the list were financial implications followed by not being psychologically ready for hearing aid, finding no benefit from amplification, the stigma attached to wearing hearing aids, discomfort from the hearing aid, and the sound quality of the hearing aids.

**Table 4:** Reasons for hearing aid returns.

Reasons	n	%
**Financial implications**	31	24.22
**The patients were not psychologically ready for hearing aids**	15	11.72
**Finding no benefit from amplification**	15	11.72
**The stigma attached to wearing hearing aids**	12	9.38
**The sound quality of the hearing aids.**	10	7.81
**Discomfort of the hearing aid**	10	7.81
**Dissatisfaction form the hearing aid**	8	6.25
**The hearing aids did not reach their expectations**	8	6.25
**Cosmetic issues that came with hearing aids**	7	5.47
**Medical aid did not cover any costs towards the hearing aid**	5	3.91
**No motivation for the use of hearing aids**	4	3.13
**Death (the hearing aids were returned by a family member)**	3	2.34
**Total:**	**128**	**100**

Table 5 below represents the physical and audiological complaints of hearing aids as described by the audiologists’. Majority of their patients reported comfort, hearing aid size, and the battery size as the most common complaint about the physical hearing aid itself. Colour and style were the least reported physical complaints of the hearing aids by the hearing aid users.

**Table 5:** Physical and audiological complaints about hearing aids.

Physical complaints	n	%	Audiological complaints	n	%
**None**	4	3.33	**Feedback**	27	13.78
**Size**	37	30.83	**Background noise**	66	33.67
**Colour**	7	5.83	**Muffled**	5	2.55
**Style**	6	5.00	**Clarity**	24	12.24
**Comfort**	40	33.33	**Echo**	27	13.78
**Battery size**	26	21.67	**Blocked**	3	1.53
			**Sharp/Tinny**	17	8.67
			**Loudness**	27	13.78
**Total:**	**120**	**100**	**Total:**	**196**	**100**

More than half of the audiologists’ reported that their patients complained about background noise as a negative factor whilst wearing hearing aids with feedback, echo and loudness being the next most common complaint. The least reported complaints from hearing aid users were muffled, blocked and sharpness/tinniness of the hearing aids.

### Trial period

Audiologists’ in this study were asked about their perceptions on whether or not audiologists’ should be offering hearing aid trial periods. The results revealed 93.68% (n = 89) believe that audiologists’ should offer hearing aid trials as part of their service-delivery to the patients. On the other hand, 6.32% (n = 6) believe that audiologists’ should not provide trials based on the following reasons:

- The hearing aid users may lose or break the hearing aid during the trial period.
- Audiologists’ do not have the opportunity to trial hearing aids in the government sector.
- Hearing aid trials were too expensive and time consuming.
- Hearing aid trials are an insurance risk to the practice.
- And lastly, as described by one participant: “the adjustment time for the brain to adapt to the new hearing aids as well as the multiple listening opportunities are not accommodated by the trial period”.

### Duration of a trial period

**Fig 2:**
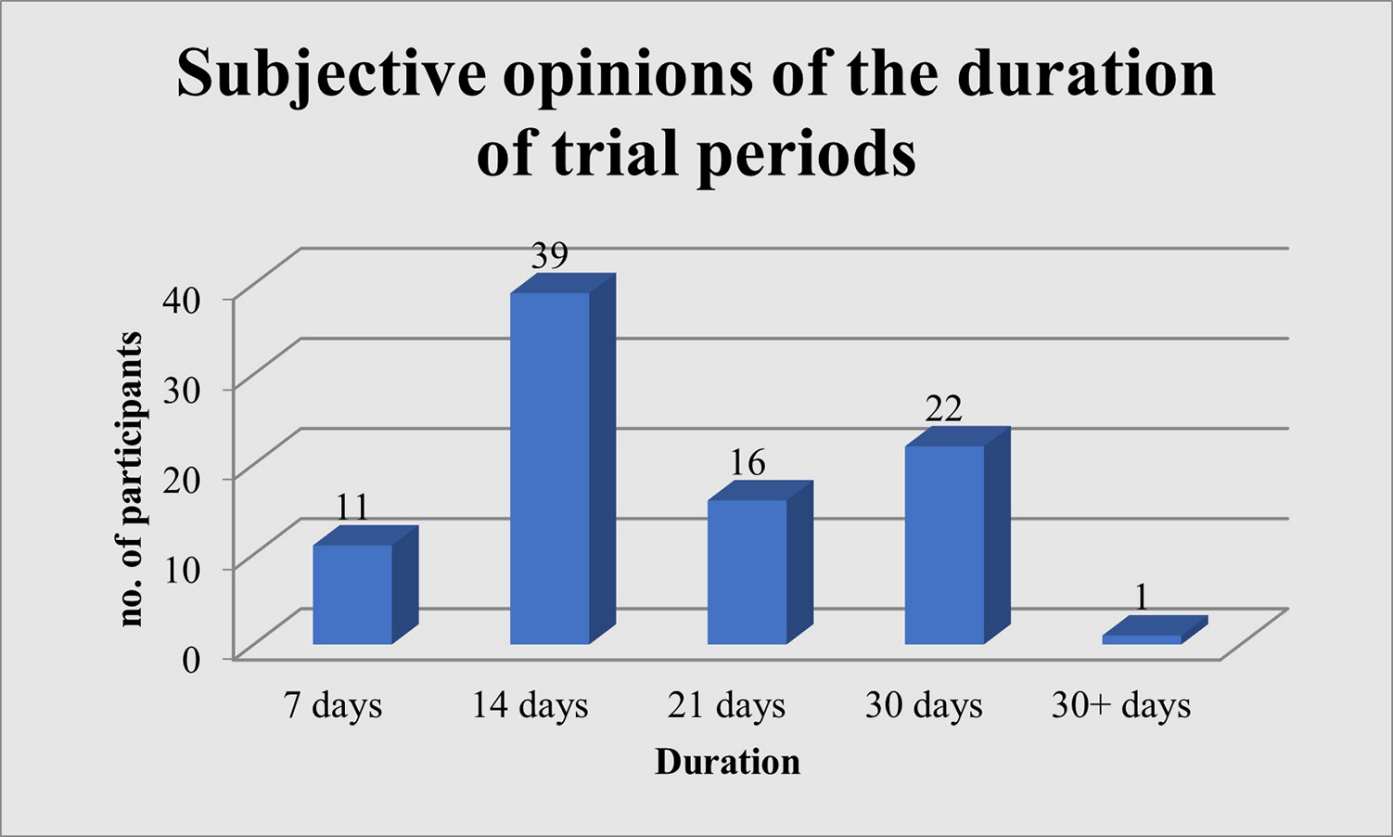
Subjective opinions of the duration of trial periods.

Audiologists’ were further asked to recommend a suitable period for hearing aid trials. Eighty-nine participants (93.68%) who felt that the trial period is a necessity made their recommendations (Fig 2). Majority of participants stated that 14 days (43.82%, n = 39) was a suitable period for a hearing aid trial, with 30 days (24.72%, n = 22) as the second suggested duration.

In establishing audiologists’ methods of hearing aid trialing practices, the results indicated that 72.63% (n = 69) of audiologists’ reported offering trialing of hearing aids as mandatory practice within their services, while 27.37% (n = 26) did not. From those who did not offer hearing trials, n = 15 reported working in a government hospital, clinic, or rural area. Reportedly, hearing aid trials were logistically impossible and thus not feasible to conduct. Some reasons for this were due to: a limited amount of available staff, long waiting lists, not being stated in the policy, not being permitted, financial implications, insurance purposes, and travel limitations from the patient’s side.

### Number of hearing aids to test per trial period

Majority of audiologists’ who offered hearing aid trials offered two different hearing aids for trialing (26.09%, n = 18) while others preferred to trial only one (17.39%, n = 12) hearing aid. Only 1.45% (n = 1) participant reported trialing 3 or more hearing aids per trial period. More than half of the participants (55.07%, n = 38) reported trialing between one to two hearing aids but it was patient-specific and circumstantial.

**Fig 3:**
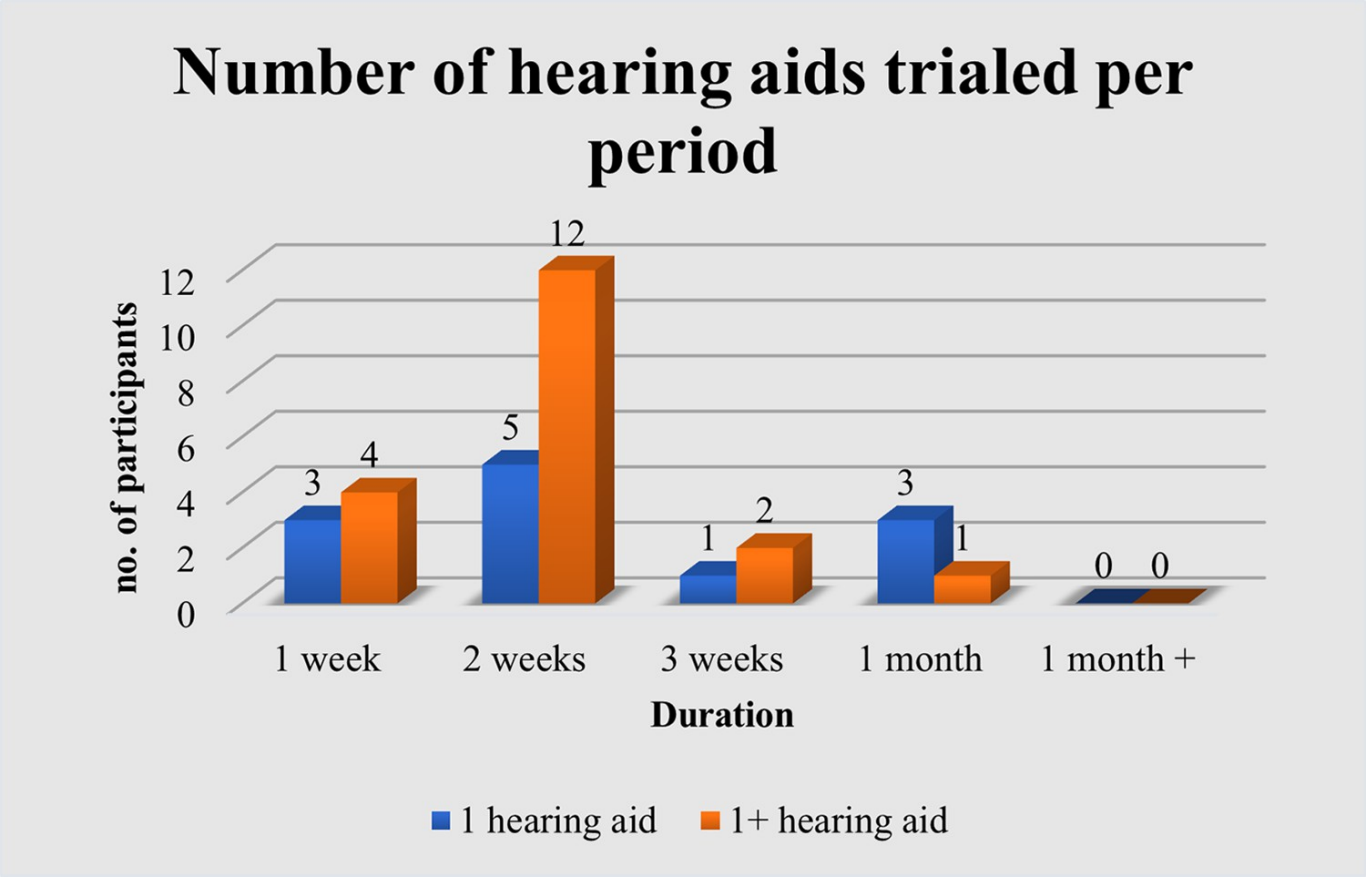
Number of hearing aids trialed per period.

To determine a suitable number of hearing aids that could be tested per trial period, the results revealed a large number of audiologists’ offered more than one hearing aid trial to their patients which can be seen in Fig 3 that depicts n = 12 participants trialing their patients for two weeks with one or more hearing aid(s). The next most common chosen duration was a one-week trial with one or more hearing aid(s).

Audiologists’ were further asked to describe their thought process when deciding to trial hearing aids. Only n = 31 participants responded to this question. The following responses were prominent:

Some audiologists’ (54.84%, n = 17) reported only allowing patients to trial hearing aids on request while 45.16% (n = 14) reported offering the hearing aid trial without it being requested by the patient. After having trialed one, two, or three hearings aids, audiologists’ reported that 77.42% (n = 24) of patients end up choosing the first hearing aid they trialed. On the other hand, 9.68% (n = 3) choose the second hearing aid and only 6.45% (n = 2) choose the third or last hearing aid that they trialed. These results indicated a preference for the first hearing aid trialed.

## Discussion

In South Africa, health care professionals such as audiologists’ and speech therapists in urbanized and densely populated provinces such as Gauteng and Western Cape tend to provide private services more than the public sector services [25]. For the less populated provinces like Limpopo, Northern Cape, Northwest and Mpumalanga, the converse is true with more health care workers in the public sector. Nationally, there are only 22% of audiologists’ and speech therapists employed in the public sector [25].

To date, the private sector in South Africa dominates the healthcare sector, attracting majority of professionals, even though the country has a larger public sector [30]. The demographic profile of audiologists’ in this study in terms of practice sector, complement the findings by two studies who discuss a shortage of key healthcare workers in the rural-urban and public-private divides within in the country [22]. Additionally, the public sector caters for approximately 84% of the population while only 16% of South Africans are served by the private sector. This inequality of services is unfortunately further worsened by unequal access to services between and within the urban and rural public sectors [25]. With this being said, fewer audiologists’ and fewer resources within the public sector limits the ability to provide access to hearing aid services [22] and therefore hearing aid trial periods.

### Hearing aid characteristics

With new advances in technology such as the digital hearing aid, there are a number of advantages over the “old school” analogue hearing aids. These include: digital feedback and noise reduction, increased comfort, smaller size, open fit designs, and speech enhancement [19]. With this, audiologists’ now have greater flexibility when choosing the best technology for their patient’s hearing needs. The majority of audiologists’ in this study supplied and fitted three or more brands of hearing aids during their fitting practices to provide their patients with a variety of options. This created the opportunity to problem solve which style or level of technology to recommend to their patients and which would benefit their personal needs the most. Furthermore, hearing aid manufacturers have provided audiologists’ with huge improvements in technology, hearing aid software, and tools—with a resultant increase in patient satisfaction [31]. Therefore, when it comes to choosing the right hearing aid the question of “who is the driving force behind the decision making?” was explored. The majority of audiologists’ in the study stated that decisions regarding the brand and style of the hearing aid is a collaborative decision between the audiologist and the patient. Only in situations where patients are uncertain do the audiologists’ make a decision on the behalf of the patient. This means that hearing aid users rely on the support, guidance and expertise of their audiologists’ when choosing a hearing aid. These results correlate with one study on success and failure factors for hearing aid prescription, where audiologist’s recommendation, followed by the price of the hearing aid, and the success of the hearing aid trial, were the factors leading to success [28]. Another study stated that considering the degree, type and configuration of a hearing loss when choosing a hearing aid increases the successful outcomes of hearing aid fittings with its useful implementation [32].

Hong et al stated that the patient and the audiologist should be equally involved in the hearing aid fitting process and that they both have responsibilities regarding hearing aid selection and fitting [31]. If each party participates and attends to their unique responsibilities, then it is more likely that the hearing aid fitting is successful.

Thus, according to the results, the researcher is in agreement with the first mentioned study [28] whereby the audiologist’s advice played an important role in hearing aid acquisition. The audiologist is therefore the driving force on the decision making when it comes to choosing the brand or style of the hearing aid for their patients.

### Return rates on hearing aids

Audiologists’ in the study stated that their patients rarely return their hearing aids once fitted, or, that one in every five returned their hearing aids at most. Audiologists’ reported the following reason for the return of hearing aid: death (the hearing aids are returned by a family member), discomfort of the hearing aid, financial implications, dissatisfaction from the hearing aid and that the hearing aids didn’t reach their expectations. Some similarities can be seen in a research study on clinical reasons for hearing aid returns in Korea from 1138 hearing aid users who were prescribed hearing aids over a three-year period. They found that ineffectiveness of the hearing aid, noise (such as feedback), over-amplification, managing and handling the devices, and lastly, financial aspects were the most common reported reasons for hearing aid returns. Despite advances in technology, 3–16% of individuals in their study returned the hearing aids over the three-year period [33].

More than half of the audiologists’ in the current study reported that their patients complained about background noise as a negative factor whilst wearing hearing aids with feedback, echo and loudness being the next most common complaints. A review of research study found that background noises and surrounding sounds were the most common complaints reported by hearing aid users [34].

### Trial period

After years of experiencing communication difficulties, which undoubtedly impacts a person’s life, the decision to try hearing aids brings about all kinds of expectations and fears. These occur due to the hearing loss, and not the hearing aids [35]. This study aimed to determine whether the availability of a trial period would influence the next step in the process, which is the decision to then actually purchase hearing aids.

As mentioned earlier, a hearing aid trial period may convince individuals to try amplification who otherwise would not do so. Almost all of the audiologist participants in this study reported that audiologists’ should be offering trial periods for hearing aids in South Africa as part of their practices. With this being said, 72.63% of audiologists’ in the study actually provide hearing aid trials to their patients. Moreover, 54,84% of these audiologists’ reported only allowing patients to trial hearing aids on request, while the other 45,16% reported offering the hearing aid trials without it being requested by the patient. These results indicated that approximately 50% of audiologists’ offer hearing aid trials to their patients as part of their daily practices. Audiologists’ who offer trials reported a higher success rate in hearing aid sales and patient satisfaction [12,27]. One study describes how the benefits of hearing aid amplification continue to increase after the initial experience with the amplified signal over a period of 6–12 weeks [36]. This means that the initial adjustment period to hearing aids is one of the most important processes to achieve successful hearing aid outcomes. With this said, not enough audiologists’ provide the opportunity for their patients to adjust to their hearing aids in order to achieve these satisfactory outcomes. Some users may not even be aware of the opportunity to trial hearing aids if not offered directly by their audiologist.

Some audiologists’ in the current study were concerned that they cannot guarantee the safe care and return of hearings aids, which poses insurance risk to their practices. Additionally, audiologists’ working in public hospitals or clinics in particular do not provide trial periods because of staff shortage, long waiting lists for hearing aids, no guarantee of return of hearing aid, and trials not being part of their standard practice. There is a shortage of staff and available resources in the rural and government sectors in South Africa which unfortunately leads to no opportunity for trial periods to be provided.

The population of individuals who have a hearing loss could be impacted from communication difficulties and a restricted social life. These negative effects of hearing loss on QoL can be improved with the use of hearing aids. The hearing aid users who adjust successfully to hearing aids typically experience higher self-esteem compared to those who do not wear their hearing aids regularly and consistently [36]. All of the audiologists’ reported that they do believe hearing aid users need time to adjust to their hearing aid(s). If this adjustment period is known, then according to the results, not enough audiologists’ are providing this service of hearing aid trials to their patients before they make their decision on which brand/style works best for them.

### Duration of a trial period

According to literature, the most common duration of a trial period is a minimum of 30 days [13,14]. However, there is no evidence-based practice to support this suggested time period. What is known is that the hearing aid manufacturing companies offer a 30-day return policy for users who wish to return their hearing aids. This period is separate to a hearing aid trialing period.

One research study complimented the current study in declaring that hearing aid users have difficulty adjusting to the hearing aids and individuals who did not wear their hearing aids regularly needed a longer time to adjust to them and should persevere during the initial period of obtaining hearing aids [5]. Audiologists’ in the current study reported that based on their experiences and practices, hearing aid users require an average of a two-week trial period or a one-month trial period before purchasing the devices. This is evident in their practices as majority of audiologists’ reported that, on average, they offer a two-week trial period.

### Number of hearing aids to test per trial period

In cases where up to three brands of hearing aids are trialed, audiologists’ reported that more than half of patients end up choosing the first hearing aid they trialed. These results indicate a preference for the first hearing aid trialed. This may be due to the users remembering the immediate benefits achieved from their first trial. Audiologists’ further stated that the average number of hearing aids they trial their patients on is two hearing aids, thus giving their patients a choice between two different brands or styles to choose from.

Hearing aids are known to improve QoL. Hearing difficulty may be reduced with an effective, well programmed, hearing aid. This will also reduce the negative effects of social and emotional interactions caused by a hearing loss [37]. With the use of an effective trial period and the correct duration and amount of hearing aids to trial, this may in turn add to the increase in QoL of hearing aid wearers as they will be able to assess the benefits of the hearing aids in all their social and working environments. In conclusion, as stated by one study “it is only the supervised and positive experience with amplification that converts them from deniers into users. In this perspective, trial periods will increase rather than decrease the number of people who purchase hearing aids” [35].

## Conclusion

Based on the findings of the current study, it can be concluded that providing hearing aid trials routinely is crucial in the rehabilitation process, while taking into account the availability of necessary resources. This study revealed 72.63% of audiologists’ provide hearing aid trials, with a smaller proportion incorporating hearing aid trials as part of their routine practice rather than solely upon request. In this study, audiologists’ agreed that offering a hearing aid trial before purchasing a hearing aid may yield desired outcomes. From an ethical standpoint, if hearing aids improve hearing aid outcomes and quality of life, audiologists’ should consider including hearing aid trials as part of their practices to enhance service delivery as healthcare professionals. While hearing aids trials demonstrate numerous benefits for users and could potentially become standard practice not only in South Africa but also internationally. As such, further research is recommended in this area.

It is also worth noting that the provision of a hearing aid trial is not only beneficial to the users but also to the audiologists’ as well. Audiologists’ stand to benefit from every hearing aid purchased and successfully fitted. Therefore, it is important to identify factors that affect hearing aid usage to implement appropriate rehabilitation strategies and ensure greater utilization of hearing aids.

Audiologists’ should play an active role in offering hearing aid trials to their patients, particularly first-time hearing aid users. Even though experienced hearing aid users may not feel confident with their hearing aids, it is important to note that technology is continuously advancing, offering improved features, sound quality, accessories, comfort and size. As such, hearing aid trials should still be offered to all hearing aid users, irrespective of their previous experience with hearing aids. There is a clear need to provide hearing aid trials to optimise outcomes for patients and ensure that they receive the most suitable and beneficial hearing aid solution.

## Limitations

A small study sample of 95 audiologists’ who conduct hearing aid trials cannot be generalized to the greater audiology population who perform hearing aid trials. Additionally, the study is limited to the South African population and thus cannot be generalized world-wide.

## Supporting information

S1 Appendix Survey for audiologists. (PDF)

## Abbreviations

WHO: World Health Organization
QoL: Quality of Life
FTC: Federal Trade Commission
FDA: Food and Drug Administration
SAHPRA: South African Health Products Regulatory Authority
HPCSA: Health Professions Council of South Africa
HREC: Human Research Ethics committee
SAAA: South African Association of Audiologists’
SASHLA: South African Speech-Language-Hearing Association
