# Supplementary material for: Hearing aid trial periods: Audiologists’ thoughts and practices in South Africa
Source: PLOS Glob Public Health. 2023 Nov 3;3(11):e0002552. doi: 10.1371/journal.pgph.0002552 (PMC10624272; doi:10.1371/journal.pgph.0002552)
Supplement: S1 Appendix — (PDF) [file pgph.0002552.s001.pdf]

Survey for audiologists

General

1. How long have you been practicing as an audiologist?

   ☐ Community service ☐ ≤5 years ☐ ≤10 years ☐ 10+ years

2. Where do you practice?

☐ Hospital ☐ private practice ☐ clinic ☐ school ☐ MDT centre ☐ other

Hearing aid characteristics

3. Do you dispense hearing aids?

   ☐ Yes ☐ No

4. Do you do provide hearing aid fittings?

   ☐ Yes ☐ No

5. Do you offer one or more hearing aid brands? (e.g Phonak, Oticon, Starkey etc)

   ☐ 1 ☐ 2 ☐ 3+

6. When fitting a patient with the hearing aid, do you choose for them or do they choose themselves?

   ☐ Patient chooses ☐ I choose ☐ Depends

   a. Please elaborate further

      ____-
      ____

Hearing aid trials

7. Do you think audiologists should be offering trial periods for hearing aids?

   ☐ Yes ☐ No

a. If yes, how long do you think is an ideal trial period?

☐ 1 week ☐ 2 weeks ☐ 3 weeks ☐ 1 month ☐ 1 month +

b. If no, why not?

____

8. Do you offer trialing of hearing aids? (If yes, continue. If not, why not?)

☐ Yes ☐ No

____

a. How many hearing aids do you allow a patient to trial?

☐ 1 ☐ 2 ☐ 3 ☐ depends

i. If you chose depends, please elaborate further

____

b. If you only offer one hearing aid to trial per period, how long is the trial period?

☐ 1 week ☐ 2 weeks ☐ 3 weeks ☐ 1 month ☐ 1 month +

c. If you trial more than one hearing aid, how long is the total trial?

☐ 1 week ☐ 2 weeks ☐ 3 weeks ☐ 1 month ☐ 1 month +

d. What made you decide to trial your patients with 1, 2 or 3 hearing aids?
Explain____
____

e. Do you only allow patients to trial hearing aids on request?

☐ Yes ☐ No

f. If you are trialing more than one hearing aid, out of the 1st, 2nd or 3rd hearing aids, on average which one do patients usually choose?

☐ 1st ☐ 2nd ☐ 3rd ☐ none

Counselling

9. Do you provide counselling to your patients pre, peri, and post fitting?

☐ Yes ☐ No

10. If yes, what topics do you cover during counselling? Please elaborate.

____
____

11. Do you provide any physical materials for patients to take home?

☐ Yes ☐ No

   a. If yes, list the types of materials given

   ____

12. Do you provide aural rehabilitation?

☐ Yes ☐ No

   a. If yes, what do you focus on during these sessions?

   ____
   ____

   b. If not, why not?

   ____

13. Do you offer follow up appointments for hearing aid orientation and counselling?

☐ Yes ☐ No

Patient satisfaction

14. On average, what are the return rates on hearing aids?

☐ 1 in every 2 patients
☐ 1 in every 3 patients
☐ 1 in every 4 patients
☐ 1 in every 5 patients
☐ Other

   a. Please specify a few reasons for hearing aid returns

   ____
   ____

15. What are the most common complaints about the physical hearing aids? (You may choose more than one).

☐ ☐ ☐ ☐ ☐

Size colour style comfort battery

☐ Other specify____

16. What are the most common audiological complaints from patients?

☐ Feedback ☐ background noise ☐ muffled ☐ clarity

☐ Echo ☐ blocked ☐ sharp ☐ loudness

17. What do you think is the most common deciding factor for your patients in purchasing their hearing aid?

☐ Characteristical features
☐ Audiological features
☐ Your recommendation
☐ Other (Specify):____

Brain maturation to hearing aids

18. Do you feel patients need time to adjust to a new hearing aid?

☐ Yes ☐ No

a. If yes, how much time do you feel is sufficient to retrain the brain from deprived sounds

☐ 1 week ☐ 2 weeks ☐ 3 weeks ☐ 1 month ☐ 1 month +

b. If no, why not?
____
____

In order to complete the second phase of this study. Im required to interview patients who have received hearing aid trials within the last 12 months or who are currently undergoing a hearing aid trial.

Would you allow me to contact and interview 1-2 of your patients chosen by you to participate in this second phase of the study? If yes please provide your name and email address below so that I can contact you.

Name and email address: ____

Thank you for participating!
